# Psychological effects of the intensified follow-up of the CEAwatch trial after treatment for colorectal cancer

**DOI:** 10.1371/journal.pone.0184740

**Published:** 2017-09-18

**Authors:** Zhuozhao Zhan, Charlotte J. Verberne, Edwin R. van den Heuvel, Irene Grossmann, Adelita V. Ranchor, Theo Wiggers, Geertruida H. de Bock

**Affiliations:** 1 Department of Epidemiology, University of Groningen, University Medical Center Groningen, Groningen, The Netherlands; 2 Department of Surgery, University of Groningen, University Medical Center Groningen, Groningen, The Netherlands; 3 Department of Mathematics and Computer Science, Eindhoven University of Technology, Eindhoven, The Netherlands; 4 Department of Surgery afd. P, Aarhus University Hospital, Aarhus, Denmark; 5 Department of Health Sciences, University of Groningen, University Medical Center Groningen, Groningen, The Netherlands; UNM Cancer Center, UNITED STATES

## Abstract

**Background:**

The aim of the study was to evaluate psychological effects of the state-of-art intensified follow-up protocol for colorectal cancer patients in the CEAwatch trial.

**Method:**

At two time points during the CEAwatch trial questionnaires regarding patients’ attitude towards follow-up, patients’ psychological functioning and patients’ experiences and expectations were sent to participants by post. Linear mixed models were fitted to assess the influences and secular trends of the intensified follow-up on patients’ attitude towards follow-up and psychological functioning. As secondary outcome, odds ratios were calculated using ordinal logistic mixed model to compare patients’ experiences to their expectations, as well as their experiences at two different time points.

**Results:**

No statistical significant effects of the intensified follow-up were found on patients’ attitude towards the follow-up and psychological functioning variables. Patients had high expectations of the intensified follow-up and their experiences at the second time point were more positive compared to the scores at the first time point.

**Conclusion:**

The intensified follow-up protocol posed no adverse effects on patients’ attitude towards follow-up and psychological functioning. In general, patients were more nervous and anxious at the start of the new follow-up protocol, had high expectations of the new follow-up protocol and were troubled by the nuisances of the blood sample testing. As they spent more time in the follow-up and became more adapted to it, the nervousness and anxiety decreased and the preference for the frequent blood test became high in replacement of conversations with the doctors.

## Introduction

Recent studies investigating follow-up strategies for colorectal cancer (CRC) patients after treatment have provided favourable evidence for more intensive follow-up protocols using the measurement of serum carcinoembryonic antigen (CEA). It has been shown that intensive follow-up protocols are associated with higher detection rate of curative recurrences and shorter detection time compared to a minimal follow-up strategies or less intensive ones [1–4]. In addition, ranging from non-significant to modest survival benefits have been reported by some studies as well [4–6]. Nowadays, such intense follow-up scheme has become guidelines for routine practice. [7,8]

The CEAwatch trial [9] is a multicentre randomized controlled trial conducted in the Netherlands between year 2010 and 2012. In this trial, the intensified follow-up protocol adheres bimonthly CEA measurements in the first three years and trimonthly CEA measurements during the fourth and fifth years combined with CT imaging. The control follow-up protocol is the Dutch care as usual follow-up guideline of which consists every 3–6 months CEA measurement and outpatient clinic visit every six months for the first three years and yearly CEA measurement and outpatient visit during the fourth and fifth year. Compared to the care as usual follow-up, the trial showed that the recurrences are detected earlier by the intensified follow-up protocol such that higher proportion of recurrences can be treated with curative intent. [9]

There is however no information with regards to the influences of the intensified follow-up protocol on the psychological aspects of patients and patients acceptance. Concerns have risen on the effects of high frequent CEA measurements and with that frequent reminders of the past disease, and the protocol that includes less frequent outpatient clinic visits and communication of test results by letters. From an implementation perspective, considering the medical benefits, the psychological outcomes should be at least comparable with the care as usual follow-up protocol.

The primary objective of the CEAwatch trial was to compare the CEAwatch follow-up scheme with the care as usual in terms of recurrence rate and detection time for the recurrences. Secondary outcomes considered were: quality of life, cost-effectiveness, and patients’ survival. The aim of the here presented analysis was to evaluate the psychological effects of the intervention follow-up protocol in the CEAwatch trial, including the impact of more frequent blood sample testing on patients’ psychological burden and worrisome of cancer, and explore patients’ experiences and expectations of the new follow-up protocol. The null hypothesis was that the intensified follow-up has no effects on patients’ attitude towards follow-up and psychological functioning. It was expected that a higher measurement frequency might on one hand give more burden and worries to patients and on the other hand might provide more reassurance. In addition, it was expected that patients would need time to adjust for the new follow-up protocol. The primary outcomes of this psychological evaluation study were patients’ attitude towards the follow-up and their psychological functioning including anxiety and depression, fear of recurrences and cancer worries. The secondary outcomes were patients’ experiences and expectations of the intensified follow-up.

## Materials and methods

### Study design

The assessments of patients’ psychological variables were performed alongside the CEAwatch trial (Netherlands Trial Register 2182, URL: http://www.trialregister.nl/trialreg/admin/rctview.asp?TC=2182, Date Registered: 26-Jan-2010). A detailed description of the trial has previously been published [9]. The CEAwatch trial is a multi-centre stepped wedge cluster randomized trial (SW-CRT) conducted between 1^st^ October, 2010 and 1^st^ October, 2012 with eleven participating hospitals from the Netherlands. Patients were recruited during the period of 1^st^ October, 2010 and 1^st^ July, 2012. The trial was approved by the Medical Ethics Committee of the University Medical Centre Groningen (METc-UMCG2010.064) on 31^st^ May 2010 and signed local feasibility declaration were obtained from all the local participating centres (Medical Ethical/Testing Committee of the Martini Ziekenhuis Groningen, Medisch Centrum Leeuwarden, Nij Smellinghe Drachten, Medisch Spectrum Twente Enschede, Meander Medisch Centrum Amersfoort, Jeroen Bosch Ziekenhuis Den Bosch, Albert Schweitzer Ziekenhuis Dordrecht, Medisch Centrum Haaglanden Den Haag, Gelre Ziekenhuis Apeldoorn, Catharina Ziekenhuis Eindhoven, and Elisabeth Ziekenhuis Tilburg). The authors confirm that all ongoing and related trials for this drug/intervention have been registered.

SW-CRT is a unidirectional design that allows the intervention to roll-out sequentially for all clusters of hospitals at different time periods of the trial [10–12]. At the beginning of a SW-CRT trial, all clusters start under the control and each cluster switches sequentially to the intervention at prespecified moments. All clusters remain under the intervention after the switch. The main motivation for adopting the SW-CRT design in the CEAwatch trial was that the computer aiding system used in the CEAwatch trial required time to be implemented at each site and SW-CRT provided logistic convenience by the phased introduction of the intervention. [12]

**Fig 1:**
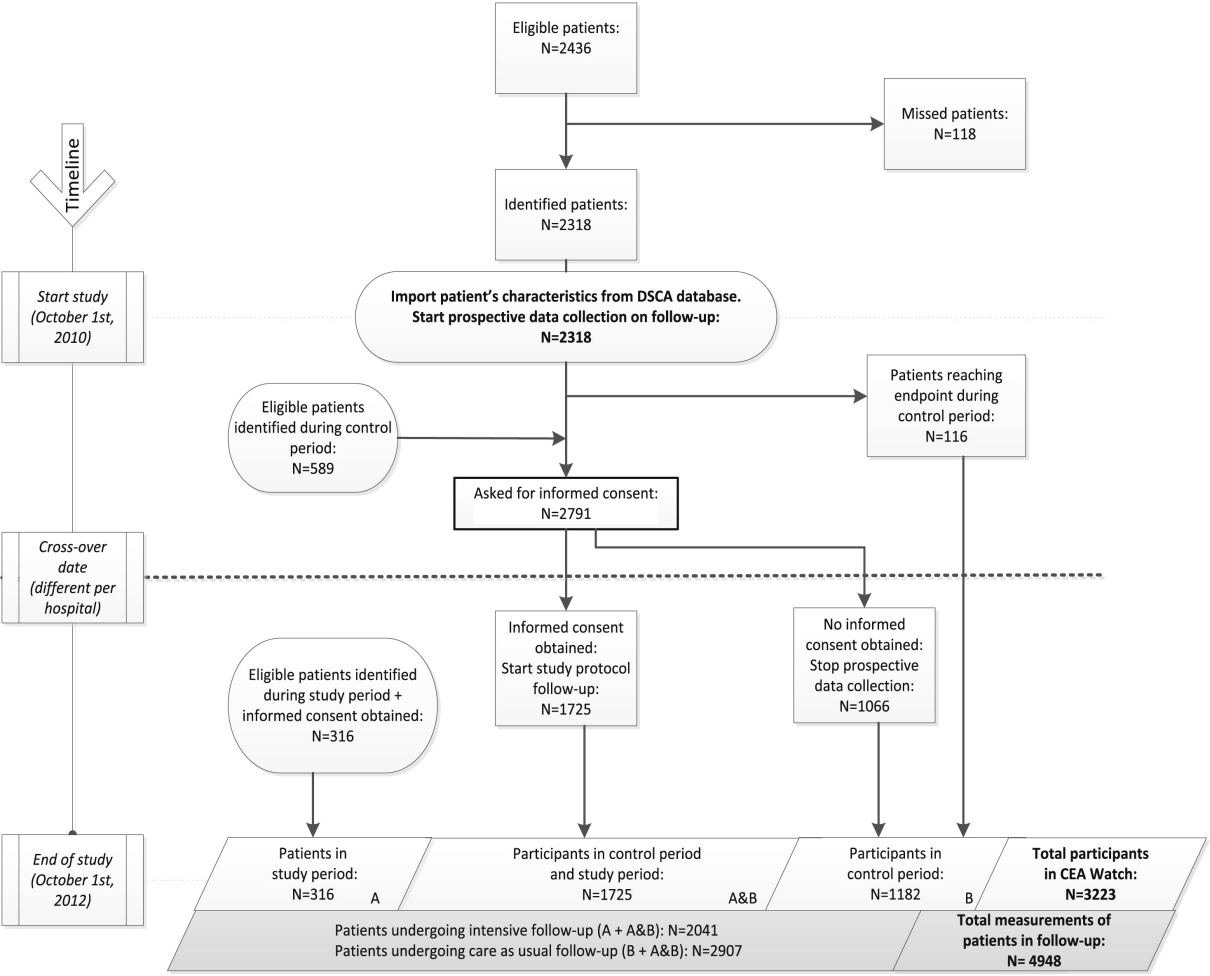
Consort diagram of the CEAwatch trial.

In the CEAwatch trial, hospitals were randomly grouped into five clusters and all clusters started with the care as usual follow-up protocol. Every three months, one randomly selected cluster switched from care as usual to intensified follow-up protocol (see Table 1). Written informed consents were obtained before the switch as required by the Medical Ethical Committee. During the trial, patients with AJCC stage I–III CRC after curative treatment were included. Patients who received adjuvant chemotherapy were eligible after cessation of the adjuvant therapy. CONSORT diagram of the CEAwatch trial is provided in Fig 1.

**Table 1:** Follow-up schedule over time, according to the stepped wedge cluster-randomized design. At day 1 of every three-monthly period a new cluster switches from the care as usual protocol (CAU) to the intensified follow-up protocol (CEA). Grey periods 1 and 2 represent the times questionnaires were sent (1st round September 2011, 2nd round June 2012).

**Cluster**	**Oct, 2010**	**Jan, 2011**	**Apr, 2011**	**Jul, 2011**	**1**	**Oct, 2011**	**Jan, 2012**	**2**
**1**	CAU	CEA	CEA	CEA		CEA	CEA	
**2**	CAU	CAU	CEA	CEA		CEA	CEA	
**3**	CAU	CAU	CAU	CEA		CEA	CEA	
**4**	CAU	CAU	CAU	CAU		CEA	CEA	
**5**	CAU	CAU	CAU	CAU		CAU	CEA	

The intensified follow-up protocol used in the CEAwatch trial adhered to every two months CEA measurements in the first three years and every three months CEA measurements during the fourth and fifth years of the follow-up. Evaluation of the rise in CEA was performed and an additional blood sample was drawn in case of CEA rise above 20% compared to the latest value, with minimum lower threshold CEA value 2.5 ng/mL. Outpatient clinic visits with imaging of thorax and abdomen were performed annually during the first three years of the follow-up. Blood test results (CEA value) including a laboratory form for the next appointment were sent to patients by automatically generated letters from a computer supporting system [13]. The care as usual follow-up followed the recommendation in the national guidelines of the Netherlands. This includes an outpatient clinic visit every six months for the first three years and annual visit during the fourth and fifth year, liver ultrasound and chest X-ray at each clinic visit, CEA measurements every 3–6 months for the first three years and once a year measurements during the fourth and fifth year.

### Data collection and questionnaires

The psychological effects of the follow-up protocol were evaluated by questionnaires sent by post. As it was not permitted to collect data prior to the obtainment of the feasibility declaration from the local centre per requirement of the primary ethical committee, it was not possible to send out questionnaires while all clusters were exposed to the control follow-up protocol. Therefore, at two time points during the trial, patients were asked to fill in the questionnaires. The first time points was September 2011, after three of the five clusters (6 of the 11 hospitals) had already switched to the intensified follow-up and the other two clusters were still in the care as usual follow-up. The second time point was June 2012, when all clusters had crossed over to the intensified follow-up and all patients had experienced the intensified follow-up (see Table 1). This had consequences of having different time between adopting intensified follow-up protocol and the psychological assessment. The durations of experiencing the new intensified follow-up protocols for patients from different clusters varied.

The questionnaires consisted of four sections: attitude towards follow-up, psychological functioning, experiences and expectations and sociodemographic data. Other disease-specific information, such as primary tumor stage, was retrieved from the CEAwatch trial.

#### Attitude towards follow-up

Patients’ attitude towards the follow-up was measured by a validated 16-item questionnaire previously developed to assess routine follow-up of colorectal cancer [14]. The questionnaire consisted of four subscales: reassurance, nervous anticipation, perceived disadvantages of the follow-up and communication (with physicians). All items were measured with Likert scales ranging from 1 to 4. Items belonging to the same subscales were combined to derive a single sum scores for each subscale, respectively. For reassurance and communication, higher scores corresponded to more positive responses, while higher score corresponded to more negative responses for nervous anticipation and perceived disadvantages.

#### Psychological functioning

The fear of recurrence was assessed by a 6-item questionnaire. From the original 3-item questionnaire used by several former studies [14,15], this instrument was extended so that it is more tailored to the trial. The English translation of the added three items can be found in Table 2. Outcomes were measured with the sum scores of the 6 items ranging from 6 to 24. A higher score indicates stronger fear. The original 3-item questionnaire had a Cronbach’s alpha of 0.75.[14] The extended version used in the present study also had high reliability (Cronbach’s alpha: 0.80) based on the current data. In addition, cancer worries were examined using the Dutch version of the validated Cancer Worry Scale [16–18], with each item using a 4-point Likert scale ranging from “never” to “almost always”. General anxiety and depression were examined by the Dutch version of the Hospital Anxiety and Depression Scale (HADS) [19]. It consisted of 14 items with 7 items for anxiety (ranging from 0 to 21) and 7 items for depression (ranging from 0 to 21). Within the HADS, a higher score meant more anxiety and depression respectively.

**Table 2:** Extended questionnaires for the fear of recurrence.

	Item	Scale
**Original**	Do you feel insecure about your health?	Not at all–Very much
	Do you think the disease might still recur?	
	Do you feel completely cured?	
**Extension**	Do you feel that the disease will certainly come back to your bowel?	
	Are you afraid that the disease will come back somewhere else than the bowel?	
	If possible, would you prefer to go to a specialist nurses?	

#### Experiences and expectations

For this part, a self-developed questionnaire was used. Patients were asked to complete 15 questions about their experiences during the intensified follow-up. If patients were still in the care as usual follow-up and had no experiences about the intensified follow-up, they were asked to answer the same 15 questions about the intensified follow-up to compare their expectations to the experiences. A 5-point Likert scale ranging from 1 to 5 was used for these items. These 15 questions are listed in Table 3.

**Table 3:** Questionnaires regarding patients' experiences of the intensified follow-up protocol.

	← More Positive				More Negative →		
1) I am satisfied with the current follow-up	Totally agree	Agree		I don’t know		Somewhat disagree	Completely disagree
2) I am afraid of blood tests§	Completely disagree	Somewhat disagree		I don’t know		Agree	Totally agree
3) I find bimonthly blood tests§:	Not stressful at all	Not stressful		I don’t know		Somewhat stressful	Very stressful
4) Bimonthly check of my blood reassures me	Totally agree	Agree		I don’t know		Somewhat disagree	Completely disagree
5) I would like my blood checked every two months	Totally agree	Agree		I don’t know		Somewhat disagree	Completely disagree
6) Transportation for intensified follow-up is a problem for me§	Completely disagree	Somewhat disagree		I don’t know		Agree	Totally agree
7) I hate to wait to turn in my blood sample§	Completely disagree	Somewhat disagree		I don’t know		Agree	Totally agree
8) I find results send by letters very pleasant	Very pleasant	Pleasant		I don’t know		Somewhat annoying	Very annoying
9) Knowing the dates of the blood testing results is of **little** importance to me§	Completely disagree	Somewhat disagree		I don’t know		Agree	Totally agree
10) I think waiting a week for the blood test results is long§	Completely disagree	Somewhat disagree		I don’t know		Agree	Totally agree
11) I think having a conversation with the doctor during visit is:	Very important	Important		I don’t know		Somewhat unimportant	Completely unimportant
12) I think frequent testing for early detection of metastases is more important than a conversation with the doctor	Totally agree	Agree		I don’t know		Somewhat disagree	Completely disagree
13) Having a conversation with the doctor once a year would be enough for me	Totally agree	Agree		I don’t know		Somewhat disagree	Completely disagree
14) I would like to know if I have a metastasis, even though I'm aware this cannot be treated for months and I have no complaints	Totally agree	Agree		I don’t know		Somewhat disagree	Completely disagree
15) I find it hard to cope with the uncertainty that the follow-up cannot guarantee the detection of the metastases§	Completely disagree	Somewhat disagree		I don’t know		Agree	Totally agree

§ The order of the options were deliberately reversed compared to the original questionnaire sent to patients so that OR>1 always indicates higher probability of being more positive.

### Statistical analyses

The outcomes on the eight subscales, namely reassurance, nervous anticipations, disadvantages, communications, HADS anxiety, HADS, depression, cancer worry scores, and fear of recurrences, were considered as the primary outcomes. Patients’ expectations and experiences were considered as secondary outcomes.

The aforementioned SW-CRT design required special attention of the secular trends in the analysis of the questionnaire data. Considering the nested structure of the design, a linear mixed model was used to assess the effects of the intensified follow-up on patients’ attitude towards the follow-up and their psychological function corrected for the secular trends. Each primary outcome was considered separately as the dependent variable in the linear mixed model. To be more specific, for each dependent variable, three types of effects were assumed, namely the time effect, the treatment effect and the differences between patients who switched from control to intervention and those who experienced intervention only for both measurement rounds. Time effect was estimated by contrasting second time measurements to the first time measurements within the group of patients who only had intervention for both rounds. Differences between the two groups of patients were assessed by comparing the two groups at the second time point. The treatment effect was estimated by contrasting two treatment groups (intensified CEA compared to CaU) at the first time but correcting for the differences between patients. The psychological effects of the follow-up protocol were also corrected for age, gender and tumor stage. Outcomes from two measurement time points were modeled as bivariate normal and hospital was considered as a random effect. (Details about the linear mixed model can be found in S1.) The p-values of the hypothesis test were adjusted for multiplicity of testing several primary outcomes [20] using the Hochberg method [21]. Since patients’ scores were not normally distributed within the attitude and psychological functioning dimensions, sensitivity analysis was conducted. These outcomes were reanalyzed with proper transformation of the outcome, namely logarithm and square root transformations. To keep the interpretation of the results simple and straightforward, the results of the linear mixed model were reported unless the sensitivity analysis would demonstrate a contradiction in conclusions. In that case, the results of the sensitivity analysis were reported instead.

To evaluate patients’ experiences and expectations of the intensified follow-up, an ordinal logistical mixed model with cumulative logit link function was applied and odds ratios were calculated for two comparisons. The first comparison is between patients’ experiences and their expectations corrected for the temporal effect. The second one is between patients’ experiences measured at the 2^nd^ time point and the experiences measured at the 1^st^ time point. The model was also adjusted for patients’ age, gender and the tumor stage. Factor analysis suggested no satisfying structural relationships among these 15 items by examining the Scree plot. Thus, the analysis was done item by item. No adjustment for multiple comparisons were made for this secondary outcome.[20]. Only the odds ratios between experiences and expectations, as well as the odds ratios of experiences between the two time points, were presented in the result section.

If patients did not complete at least 80% of the items within certain subscales or dimensions, the score of this subscale/dimension was considered missing. Missing data was considered to be missing at random (MAR) and no special treatment for missing data was needed since inferences with maximum likelihood (used in both the linear and generalized mixed models) are still valid under this assumption. Statistical analyses were performed with SAS^®^ statistical software, version 9.4. Linear mixed models were fitted using PROC MIXED and generalized linear mixed models were fitted using PROC GLIMMIX.

## Results

### Patient characteristics and response rate

On November 1st, 2011, total of 2,016 patients participated in the CEAwatch trial, and received the questionnaires. A total of 1,591 patients (78.9%) returned the questionnaires. On May 1st, 2012, total of 1,848 patients participated in the CEAwatch trial, 1556 (84.2%) of them returned the questionnaires. Patient characteristics of the two rounds are given in Table 4. During the first round, 820 (51.6%) of them participated in the care as usual follow-up and 770 (48.4%) were in the intensified follow-up (1 missing). At second round, all patients (2 missing) were in the intensified follow-up (Table 4). Among all patients, 1162 of them participated in both rounds of questionnaires. Summary of patients’ experiences and expectations questionnaire is available in S1 Table.

**Table 4:** Patient characteristics and summary of primary outcome scores for the first round and second round evaluations.

	Round 1 (n = 1591)	Round 2 (n = 1556)
**Age: median (range)**	68 (26–94)	68 (29–93)
**AJCC stage**^**1**^		
I	422 (27.80%)	433 (29.94%)
II	595 (39.20%)	572 (39.56%)
III	501 (33.00%)	441 (30.50%)
**Gender**^**2**^		
Female	685 (43.11%)	621 (40.01%)
Male	904 (56.89%)	931 (59.99%)
**CEA follow-up**^**3**^		
Intervention	770 (48.43%)	1554 (100.00%)
Control	820 (51.57%)	0 (0.00%)
**Attitude towards follow-up**	median (range)	median (range)
Reassurance	13 (4–16)	13 (4–16)
Nervous anticipation	7 (5–20)	7 (5–18)
Perceived disadvantages	4 (3–11)	4 (3–11)
Communication	13 (4–16)	13 (4–16)
**Psychological functioning**	median (range)	median (range)
Fear of recurrence	12 (6–24)	12 (6–22)
HADS: Anxiety	3 (0–21)	3 (0–21)
HADS: Depression	2 (0–20)	1 (0–20)
Cancer worries	13 (8–31)	13 (8–31)

^1^ Missing 73 for round 1 and missing 110 for round 2

^2^ Missing 2 for round 1 and missing 4 for round 2

^3^ Missing 1 for round 1 and missing 2 for round2.

#### Primary outcomes

The estimations for the psychological effects on patients’ attitude towards follow-up and psychological functioning of the intensified follow-up protocol and time periods differences are shown in Table 5. No statistical significant effects of the intensified follow-up were found on patients’ attitude towards the follow-up. Furthermore, there were no significant differences on anxiety and depression, fear of recurrences and cancer worries between the intensified follow-up protocol and care as usual follow-up. Comparing between two time points, no statistically significant temporal differences were found for all subscales.

**Table 5:** Estimates and 95% confidence limits of follow-up protocol effects and secular trends from linear mixed model for patients' attitude towards the follow-up and psychological functioning.

	Intensified follow-up vs. care as usual				Time trends			
	*Estimates*	*95% CL*		Adjusted p-value^*^	*Estimates*	*95% CL*		Adjusted p-value^*^
**Reassurance**	0.1202	-0.4504	0.6909	0.64	-0.2347	-0.5310	0.0617	0.42
**Nervous anticipation**	0.5738	-0.2669	1.4146	0.64	-0.5423	-0.9690	-0.1156	0.12
**Perceived disadvantage**	0.2544	-0.2815	0.7904	0.64	-0.2153	-0.4880	0.0574	0.42
**Communication**	0.2365	-0.5618	1.0348	0.64	-0.3121	-0.7211	0.0967	0.42
**HADS: Anxiety**	0.6135	-0.0490	1.2759	0.56	-0.4348	-0.7925	-0.0771	0.12
**HADS: Depression**	0.3258	-0.4189	1.0706	0.64	-0.1461	-0.5319	0.2396	0.42
**Cancer worries**	0.2510	-0.7325	1.2346	0.64	-0.2275	-0.7319	0.2768	0.42
**Fear of recurrence**	0.2229	-0.8381	1.2838	0.64	-0.2264	-0.7651	0.3122	0.42

* Adjusted p-values were calculated according to the Hochberg method for multiple comparison adjustment.

#### Secondary outcomes

**Fig 2:**
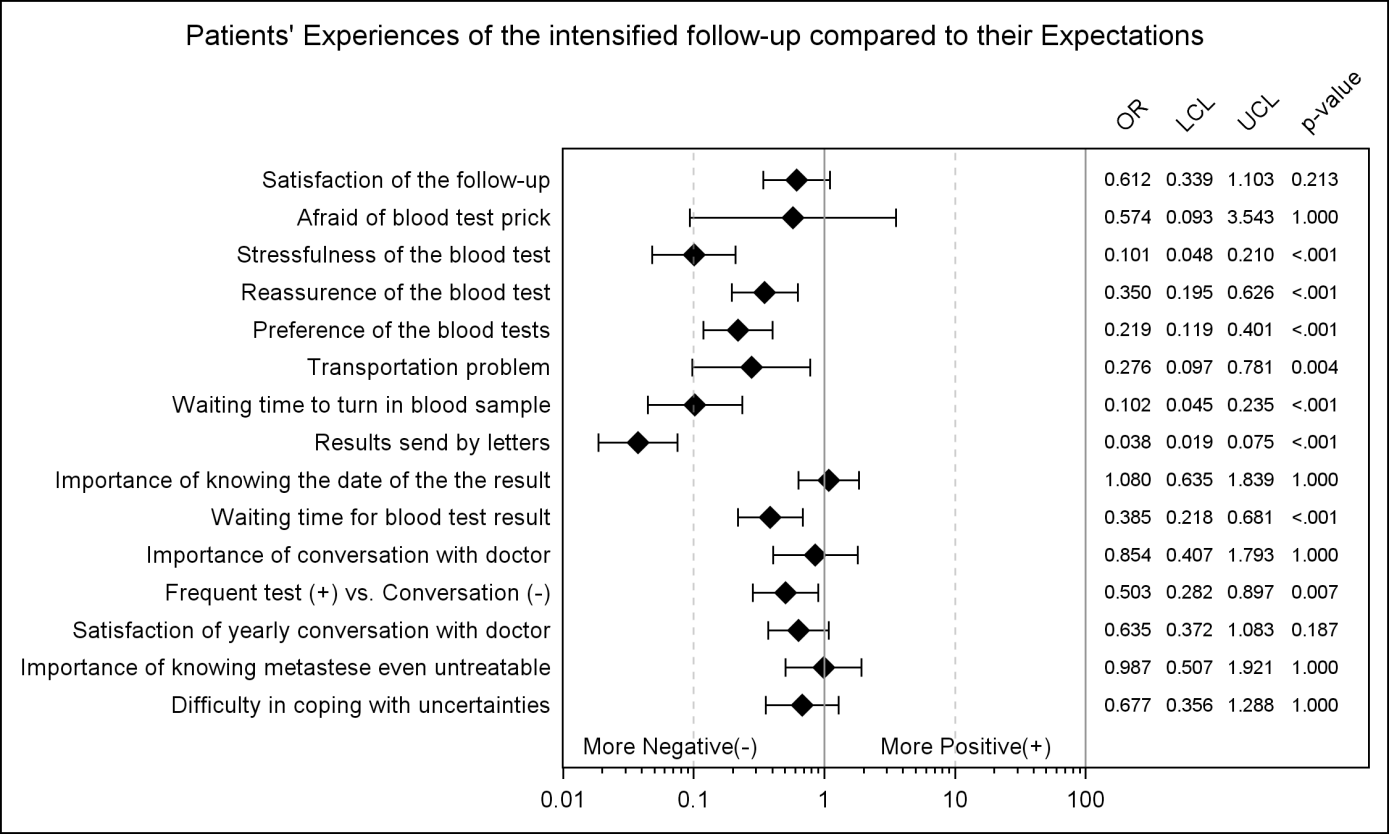
Patients experiences of the intensified follow-up compared to their expectations.

The comparisons between patients’ experiences and expectations are shown in Fig 2. In general, comparing patients’ experiences in the intensified follow-up to their expectations, the responses were towards the negative end of the spectrum. Particularly, patients expressed that the stress of the blood test was higher than they expected (OR: 0.10, 95% CL: [0.06, 0.16], p-value: <0.001) while they were less reassured by it (OR: 0.35, 95% CL: [0.24, 0.52], p-value: <0.001) and the preferences of the blood tests were not in favour of the intensified follow-up (OR: 0.22, 95% CL: [0.15, 0.33], p-value: <0.001). In addition, the inconveniences of the blood tests such as transportations (OR: 0.28, 95% CL: [0.14, 0.55], p-value: 0.0003), waiting time to turn in a blood sample (OR: 0.10, 95% CL: [0.06, 0.18], p-value: <0.001) and results sent by letters (OR: 0.04, 95% CL: [0.02, 0.06], p-value: <0.001) were less appreciated.

**Fig 3:**
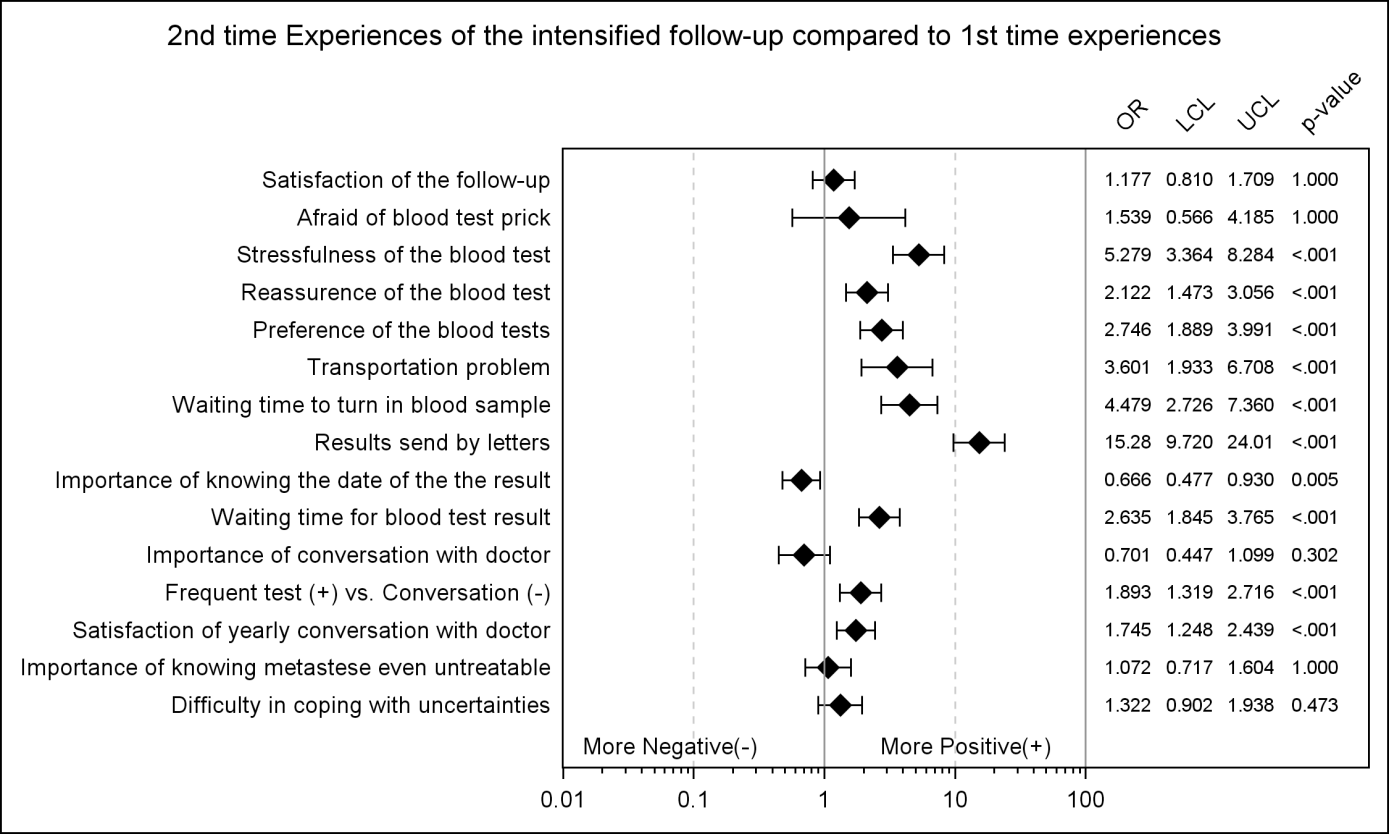
Patients’ 2^nd^ time experiences of the intensified follow-up compared to their 1^st^ time experiences.

In the comparisons between patients’ second experiences and their first time experiences, the responses at the second time were more positive than the one at the first time as shown in Fig 3. At the second time points, patients had statistically significant higher probability to give a more positive response. Specifically, patients were more positive about all the items that did not meet with expectations in the previous comparison. Blood tests were less stressful (OR: 5.28, 95% CL: [3.91, 7.13], p-value: <0.001) and provided more reassurance (OR: 2.12, 95% CL: [1.66, 2.71], p-value: <0.001) at the second time point compared to their first time experiences. Preferences of the blood test became higher (OR: 2.75, 95% CL: [2.14, 3.52], p-value: <0.001) and the frequent tests were more preferred in replacement of having conversation with the doctors (OR: 1.89, 95% CL: [1.49, 2.41], p-value: <0.001). Satisfaction of yearly conversation with the doctors became higher as well (OR: 1.75, 95% CL: [1.40, 2.18]. p-value: <0.001) with the importance of the conversation with the doctors decreased (OR: 0.70, 95% CL: [0.52, 0.95]. p-value: 0.02). Furthermore, patients felt easier coping with uncertainties of the test (OR: 1.32, 95% CL: [1.03, 1.71], p-value: 0.03)

#### Sensitivity analysis

The hypothesis tests of the linear mixed model could be affected by the skewed residual of the data. For reassurance subscale, the conditional residual was negatively skewed and the dependent variable itself was first converted to positive skewness and then logarithm-transformed. The estimations after the transformation (both treatment effect and time effect) were more towards the null and were consistent with the estimations of the linear mixed model. For nervous anticipation and cancer worry subscale, direct logarithm transformations were applied respectively. The treatment effect remained non-significant and the time effect remained significant for nervous anticipation. Both effects were shifted towards the null for cancer worry subscale. For both HADS subscales, square root transformations were used and the results remained the same. The rest of the subscales were normally distributed. Detailed sensitivity analysis results are available in the S2 Table. To conclude, the results of the sensitivity analysis agreed with the linear mixed model and the estimations presented were accurate enough to be clinically meaningful.

## Discussion

In the CEAwatch trial, an intensified follow-up protocol was compared to the Dutch care as usual follow-up guideline. The major differences in the intensified follow-up protocol relevant to the discussion of the present study was that the frequency of outpatient clinic visit during the first three years of the follow-up were reduced and in replacement was a more intensive CEA measurements scheme.

The effects of the intensified follow-up protocol for CRC patients after surgery in the CEAwatch trial were evaluated with regards to patients’ psychological variables. No statistical significant effects were found on patients’ attitude towards the follow-up and psychological functioning. For patients’ psychological functioning, no proof of increased burden or improvement was observed comparing the intensified follow-up protocol to the care as usual follow-up protocol.

Comparisons between patients’ experiences and expectations resulted in more negative responses for patients’ experiences which indicate that the expectations of the new follow-up protocols were high. On the other hand, by analysing the experiences at two different time points, we found that the responses became more positive later in time. Especially, patients responded more positively to blood test including reassurance, stressfulness and preference. This is in accordance with the results from the primary outcome that no decrease in reassurance were observed since it has been shown that patients are reassured by outpatient clinic visits and having conversation with the doctors [14]. From the present study, one may deduce that the frequent blood test compensated for less frequent clinic visit in the intensified follow-up protocol in terms of reassurance. In addition, patients’ responses to the inconveniences of the blood tests were improved with time as well.

It has been mentioned that follow-up may remind patients of their cancers and possible relapsing of malignant disease [14]. However, even with more frequent blood tests, patients’ cancer worries and fear of recurrences did not increase, nor did the HADS anxiety scores., Though it is expected that patients were more nervous and anxious about the new follow-up protocol as they were inexperienced with this new strategy, no significant differences were found between the earlier assessment time point and the later time point. On the other hand, from the exploratory analysis results of patients’ experiences and expectations, it was indicated that patients’ preferences with the proposed intensified follow-up protocol increased as they became more familiar with the protocol.Currently, limited information is available regarding the impact of follow-up protocols on patients’ quality of life and psychological functioning [14,22] from the literature. The FACS study also planned to investigate the quality of life and satisfaction of care of the colorectal cancer follow-up and the results have not been published yet. The presented study with large sample size and high response rate, provided such information for the state-of-art post-treatment follow-up protocol. It should be noted that the statistical method used in the present study explicitly assumed no treatment-period interaction. In case treatment-period interaction was present, the estimator would be biased and could lead to an opposite conclusion on treatment effect. Thus our results should be viewed under the assumption of no treatment-period interaction effect. Due to the restricted policies from the medical ethical committee and the required time for collecting questionnaires by post, collecting data from more periods was infeasible. As such, it prohibits the possibility to investigate and verify the assumption of no treatment-period interaction which we originally planned for. Meanwhile, the results of the secondary outcome should be interpreted with caution since for this study relevant questions were formulated and these were analyzed item by item. The purpose was to provide a qualitative insight in patient’s expectations and experience, tailored to the features of the intensified follow-up protocols used in the CEAwatch trial. In our opinion, it is sufficient enough to provide indirect evidence on the general trends of patients’ experiences with regards to the intensified follow-up and is in agreement with the primary outcomes. In addition, doubts have been raised as to the validity of the HADS. It is recommended not to use this instrument anymore for future study. However, the questionnaires were already used by then.

In conclusion, the intensified follow-up protocol posed no adverse effects on patients’ attitude towards the follow-up and psychological functioning. In general, patients had high expectations of the new follow-up protocol and were troubled by the nuisances of the blood sample testing at the start of the new follow-up protocol. As they spent more time in the follow-up and became more adapted to it, the preference for the frequent blood test became high in replacement of conversations with the doctors.

## Supporting information

S1 Table Summary of secondary outcome scores for the first round and second round evaluations. (XLSX)

S2 Table Results of the sensitivity analysis. (XLSX)

S1 File Descriptions of the linear mixed model used in the analysis of the primary outcomes. (DOCX)

S2 File Consort checklist. (DOCX)

S3 File Study protocol. (DOC)

S4 File Dataset of the study. (SAS7BDAT)
