# Supplementary material for: Psychological effects of the intensified follow-up of the CEAwatch trial after treatment for colorectal cancer
Source: PLoS One. 2017 Sep 18;12(9):e0184740. doi: 10.1371/journal.pone.0184740 (PMC5603155; doi:10.1371/journal.pone.0184740)
Supplement: S1 File — (DOCX) [file pone.0184740.s003.docx]

# **S1. Statistical model descriptions**

The following model was fitted to each of the primary outcomes of the study separately:

$$Y_{ijk}=a_{i}+b_{k(i)}+Age_{ik}+Gender_{ik}+AJCC_{ik}+Group_{ij}+e_{ijk},$$

with $Y_{ijk}$ as the outcome of kth patients at jth round from ith hopistal, $a_{i}$ a normally distributed random effect of the hospital i, $b_{k(i)}$ a normally distributed random effect of patient k nested within hospital i, $Age_{ik}$ a continuous variable representing patient’s age when the measurement took place, $Gender_{ik}$ a categorical variable for patient’s gender (F=female, M=male), $AJCC_{ik}$ a categorical variable for patient’s AJCC tumour stage of the primary tumour (1=Stage I, 2=Stage II, 3=Stage III), $Group_{ij}$ a categorical variable that was used for contrasting both the difference between the follow-up protocols and the difference between the two time points. For patients at the first time point experiencing the CAU follow-up protocol, the Group variable was coded as 1, and for patient at the first time point already exposed to the intensified CEA follow-up protocol, the Group variable was coded as 2. Furthermore, at the second time point, patients who were under CAU and under intensified CEA protocol in the previous round was coded as Group 3 and Group 4, respectively.

Treatment effects were estimated by the following contrast:

Treatment Effect = (Group 3 – Group 1) – (Group 4 – Group 2),

Period effects were estimated by the following contrast:

Period Effect = Group 4 – Group 2.

Group or cohort differences at follow-up were estimated with the contrasts:

Cohort Effect = Group 4 – Group 3.
