# Supplementary material for: Psychological effects of the intensified follow-up of the CEAwatch trial after treatment for colorectal cancer
Source: PLoS One. 2017 Sep 18;12(9):e0184740. doi: 10.1371/journal.pone.0184740 (PMC5603155; doi:10.1371/journal.pone.0184740)
Supplement: S3 File — (DOC) [file pone.0184740.s005.doc]

**Computer supported CEA surveillance after curative treatment of colorectal cancer**

**Protocol identification number/code**

**Netherlands Trial Register: NTR 2182**

**ABR number: NL31410.042.10**

**ZonMW project number: 171002209**

**Study coordinator: Drs C Verberne**

**Writing committee:**

***Dr. K. M. Vermeulen***

***Drs. I. Grossmann***

***Dr. K. Havenga***

***Dr. J.M. Klaase***

***Dr. A. Renehan***

***Dr. G.H. de Bock***

**Principal investigator:**

***Prof. dr T. Wiggers***

***University Medical Center Groningen***

***Department of Surgical Oncology***

***PO Box 30.001***

***9700 RB Groningen***

***Tel: + 31 50 3612317***

***e-mail: t.wiggers@chir.umcg.nl***

**Sponsor:**

***The Netherlands Organisation for Health Research and Development (ZonMw)***

**Independent physician**

***Prof. dr. J. Plukker***

**TABLE OF CONTENTS**

1. SUMMARY

2. INTRODUCTION AND RATIONALE

2.1 Introduction and background

2.2 Follow-up

2.3 Carcinoembryonic antigen

2.4 CEA watch

2.5 Imaging

2.6 Previous studies by our group

3. OBJECTIVE

4. ENDPOINTS

4.1 Primary endpoint

4.2 Secondary endpoints

5. STUDY DESIGN

6. STUDY POPULATION

6.1 Population (base)

6.2 Inclusion criteria

6.3 Exclusion criteria

6.4 Sample size calculation

7. TREATMENT PERIODS

7.1 Control period

7.2 Intervention period

7.2.1.Procedure when CEA crosses the threshold value (see flowchart, addendum 2)

8. METHODS

8.1 Study parameters

8.1.1. Main parameter/endpoints

8.1.2. Secondary study parameters/endpoints

8.1.3. Other study parameters

8.2 Study procedures

8.3 Withdrawal of subjects

8.4 Replacement of individual subjects after withdrawal

8.5 Follow-up of subjects withdrawn from treatment

8.6 Premature termination of the study

9. SAFETY REPORTING

9.1 Section 10 WMO event

9.2 Adverse and serious adverse events

9.3 Follow-up of adverse events

9.4 Safety monitoring board (DSMB)

10. STATISTICAL ANALYSIS

11. ETHICAL CONSIDERATIONS

11.1 Regulation statement

11.2 Recruitment and consent

11.3 Benefits and risk assessment, group relatedness

11.4 Compensation for injury

11.5 Incentives

12. ADMINISTRATIVE ASPECTS AND PUBLICATION

12.1 Handling and storage of data and documents

12.2 Amendments

12.3 Annual progress report

12.4 End of study report

12.5 Public disclosure and publication policy

13. REFERENCES

**1. SUMMARY**

**Title**: Computer supported CEA surveillance after curative treatment of colorectal cancer

**Aim of the study**: The aim of the current study is to develop an evidence-based guideline with standard diagnostic tools (CEA and CT scan) that results in a (cost-) effective recommendation which is applicable for all hospitals in the routine follow-up after curative treatment of colorectal cancer.

**Study design**: In the intervention group follow-up, care consists of bi-monthly CEA measurements starting three months after surgery, a yearly outpatient clinical visit and CT imaging after one and two years. Patients will be closely monitored in an outpatient setting by a frequent testing of CEA, supported by a computer-based system (CEA watch). The patients will be asked to assess their CEA value every 8 weeks. The computer-based system allows us to closely monitor the blood values and to communicate the results to the patient as soon as possible (by e-mail and/or p-mail). In case of two consecutive significant rises in CEA values, extra imaging diagnostics will be performed. In the control group the intervention is follow-up care as usual according to the Dutch national guideline. The starting point of the new regimen will be randomised among the participating clinics.

**Endpoints**: Primary outcome: increase of 15 percent of radical resected metastases in patients as expressed as the percentage of total recurrences. Secondary outcomes: overall survival time cancer specific survival time, time to recurrent disease, quality of life, and costs related to diagnostics.

**Statistics**: Increase in percentage of resected metastases from 10-25% needs 115 patients with metastases in both groups. With an expected recurrence rate of 25%, 460 patients per group are needed. After correction factor about 800 patients per group are needed.

In the economic evaluation costs of both follow-up strategies will be assessed and compared. In addition cost-utility analysis will be conducted based on Quality Adjusted Life Years (QUALY’s).

**Study duration**: The study will be conducted in three years. After a start up period of six months all eligible patients will be identified and registered. Three months later every three months a new pair of hospitals will be randomized to start the new schedule of follow-up and all registered patients will start with the intensive schedule. The last three months will be used for analysis of the data.

**2. INTRODUCTION AND RATIONALE**

*2.1 Introduction / background*

The incidence of metachronous metastases after curative colon and rectum resections is approximately 30-50%, with an increasing incidence dependent on the initial stage. Predominant sites of first recurrence in the population with recurrent disease are the liver (± 40%), lung (± 30%) and local recurrence (± 25%) (Kobayashi). These types of recurrent disease can be eligible for curative resection, which is now feasible in an estimated 10-30%. The increase in survival after metastasectomy for resectable recurrent disease is high, with a 5 year overall survival after metastasectomy of approximately 30% for all three types of recurrent disease (Goldberg, Bentrem, Pfannschmidt). It is predominantly dependent on achievement of a R0 resection and the number of metastases, the last probably corresponding to tumor behaviour.

*2.2. Follow-up*

After curative treatment of colorectal cancer, patient surveillance is recommended to detect curable recurrent disease or new primary tumors in an early phase. There was a statistically significant increase in feasibility of intended curative resection of recurrent disease in 5 prospective randomised studies and in the 5 year overall survival in 4 of the 5 studies. Based on the literature, both the Dutch guideline (CBO 2008), the Canadian guideline and the US guideline (ASCO 2005) propose a high intensity program of regular office visits, imaging studies (CBO: liver ultrasound, Canada: liver imaging, ASCO: Computed Tomography (CT) chest-abdomen) and CarcinoEmbryonicAntigen (CEA) tests. Imaging is advised since not all recurrences are detected with an early CEA raise. The value of physical examination in detecting recurrent disease is low (Borie, Sugarbaker).

*2.3. Carcinoembryonic antigen (CEA)*

Carcinoembryonic antigen (CEA) is a serum tumor marker used in follow-up of colorectal cancer. It is a protein expressed at the surface of endodermal cells during embryological development, where it has a role in cell-to-cell adhesion. In adults, it is expressed in malignancies and benign inflammatory disease of endodermally derived organs, such as the small and large bowel, pancreas, lung and thyroid. Smoking and other malignancies such as breast and ovarian cancer can increase serum CEA as well. Gold and Freedman discovered it as one of the first serological tumor markers in 1965. Staab developed the basis for it’s clinical application around 1980, after which it was introduced in daily clinical practice for colorectal cancer. The advised frequency of CEA testing in guidelines is ambiguous. The Dutch guideline advices CEA measurements every 3 to 6 months for the first 3 years, and every 6 months in year 4 and 5. The US guideline for CEA advices every 3 months for at least 3 years. Furthermore, no advice is given on the interpretation of CEA results. The key to an effective use of CEA is frequent assessment and an interpretation of test results based on the absolute value as well as on the trend in CEA increases (if present). In this way both sensitivity and specificity of CEA testing may be increased. (Steele, Boey, Staab, Grossmann (b)). Several authors mention the lack of compliance to frequent CEA measuring, especially in the elderly patient (Cooper). However, in our single-centre trial with intensified CEA measurements, compliance of patients was high. (Verberne, manuscript in preparation) and major part of the problem might be the doctor’s low compliance to the guidelines. As a result, in daily practice a significantly lower frequency of CEA was generally performed; causing a much less effective result of CEA testing (Grossmann(a), Graham, Steele, Minton, Grossmann (c), Spratlin). With the availability of a computer support system this problem is mostly solved.

*2.4.* *CEA watch*

The innovative element of the study is the introduction of CEA watch. This intranet based program based on open source software: MySQL database, PHP scripting and java. It automatically presents new CEA values of enlisted patient to the interpreting doctor. Normal results of CEA can immediately be sent by e-mail to the patient. A suggestion for a date for a new CEA check is made based on available information. In the case of a rise in CEA the patient will be called and appropriate steps will be made. All results will be sent to the patients by postal mail as well, with a laboratory form for the next CEA check. For this next CEA check the patient can report to the hospital laboratory at a date or time by his own choosing. If a patient seems to forget a CEA check, CEA-watch will issue a reminder. CEA-watch has several advantages for the patient and for the clinic. Outpatient clinic visits are reduced. The patient will come to the outpatient clinic only once a year if the results are normal. This will reduce concerns to the outcome of the disease by the patient and his family, as every outpatient clinic visit is a renewed confrontation with the disease. Furthermore, it will reduce the workload for the outpatient clinic. Compliance to the schedule of frequent testing is achieved by constant monitoring of every individual patient. Patient satisfaction is expected to increase due to the early availability of the result and the reduction of waiting time in the hospital since the patient only has to come to the laboratory at a moment chosen by him. The relevant clinical outcome is the increase in the number of radical resected metastases (lung, liver and peritoneal cavity) with a subsequent increase in cancer-specific survival.

*2.5. Imaging*

CT scanning may be superior to ultrasound in terms of sensitivity, specificity and inter- and intraobserver reliability, but it is more expensive than ultrasound and related to radiation-based health hazards. As the optimal frequency for imaging is not known, it may be expected that the use of ultrasound and CT will increase as will the logistical and financial consequences of its use without known efficacy.

*2.6. Previous studies by our group*

In a web-based survey of follow-up after treatment of colorectal cancer, which was sent to all registered Dutch general surgeons, a reply from 246 surgeons treating patients for colorectal carcinoma in 105 out of 118 hospitals was received (response rate 91%; Grossmann (a)). The generally accepted follow-up protocol consisted of CEA measurements every 3 months in the first year and six-monthly thereafter, and ultrasound examination of the liver every 6 months. Nearly all surgeons (92%) were willing to participate in a new study of follow-up protocol. In a study of literature investigating why CEA failed to prolong survival in clinical practice, several factors that can be affected were found; both in method (e.g. threshold interpretation), timing, imaging techniques following abnormal results and logistics (Grossmann (b)). Based on these findings a multicenter prospective phase II trial, investigating the optimal frequency and interpretation of CEA in follow-up is ongoing (OptCEA trial; METc 2207/015 UMCG, METC P07-034 MST.). Preliminary data (manuscripts in preparation) confirm that the feasibility of a follow-up study without a computer support system is difficult. This trial is one of the bases of our study, because with the results of this trial we were able to define the best flowchart to optimize the protocol and construct a large multi-center prospective study design. However, a phase III trial including the cost-effectiveness of the new follow-up scheme is not performed yet. The abdominal CT has been proven highly valuable. Routine staging with chest CT has not led to improvement in survival and neither to a better quality of life. A staging chest CT should not be done as a routine procedure in patients with either colon or rectal carcinoma. A concurrent study has been undertaken investigating the psychological effects of intensive follow-up and optimal communication towards the patient (Reijnen). A computerized follow-up system to support the more complex organisation of the study protocol has been developed and already implemented in the UMCG. Initial experiences are good. Most patients and doctors accept this new method and no practical problems have occurred at this time. Preliminary data show a large decrease in workload for the doctors and much convenience with patients because of the earlier communication of the blood results. In the Netherlands a prospective continuous data measurement (audit) of all colorectal cancer patients has started January 1^st^ 2009 (http://www.dsca.nl). The principal investigator is part of the scientific committee and this data base can be used as a reference for the present clinical study.

**3. OBJECTIVE**

The aim of the current study is to develop an evidence-based guideline with standard diagnostic tools (CEA and CT scan) that results in a (cost-) effective recommendation which is applicable for all hospitals in the routine follow-up after curative treatment of colorectal cancer. All patients after a curative resection of the primary tumour (including a radical resection of synchronous metastases) and fit to undergo major surgery again if needed are eligible for this study.

**4. Endpoints**

*4.1. Primary endpoint*

The increased percentage of patients with intended curative metastasectomy of liver- and lung metastases from colorectal carcinoma in the intensified follow-up schedule.

*4.2. Secondary endpoints*

1) Overall survival, disease specific survival, disease free survival

2) Calculation of the optimal threshold values and measurement frequency of CEA

3) The correlation between CEA and helical CT abdomen/thorax: specifically whether the metastasis can be localized when CEA values suggest metastatic disease.

4) Evaluation of the effects of the follow-up protocol: psychological effects and logistic effectuation

**5. STUDY DESIGN**

This trial is a randomized national multicentre study comparing intensified and conventional follow-up schedule for patients after curative treatment of colorectal cancer. The inclusion is during maximal 2.5 years or until 2000 patients are included. Total expected duration 3 years. Patients may enter during their first three years after curative treatment[[1]](#footnote-2) of colorectal cancer.

The study is constructed with the stepped-wedge design. This implies a randomization at hospital level. The surgeons in a hospital control all their eligible patients according to the care as usual. At the moment determined by the randomization, all included patients will change to the intensified study follow-up protocol. As a result, the early-randomized hospitals have a large amount of patients in the study group and a small amount of patients in the control group, where in the late-randomized hospitals it is the other way around. Randomization will take place on July 1^st^, 2010 and randomization will be performed per two hospitals. The firstly randomized hospitals start with the intensified follow-up scheme on January 1^st^, 2011 and since ten hospital will participate, the last two hospitals start with the intensified follow-up scheme at January 1^st^, 2012.

**6. STUDY POPULATION**

## *6.1. Population (base)*

Patients with stage II, III, and IV colorectal carcinoma after curative resection (R0 resection); this includes all new patients and patients currently in follow-up, operated within three year before start study.

## *6.2. Inclusion criteria*

Patients with stage II- III –IV colorectal carcinoma after curative resection (R0 resection)

- All new patients

- Patients currently in follow-up, operated within three years before start study

- All patients need to be above 18 and capable of understanding the Dutch language

## *6.3. Exclusion criteria*

Patients with other malignancies except basocellular carcinoma of the skin

Patients not medically fit for metastasectomy

Patients with diagnosed syn- or metachronous incurable metastases at time of start study

No written informed consent

## *6.4. Sample size calculation*

Given current usual care with pre-operative staging with CT of the thorax and abdomen and neo-adjuvant therapies, the expected occurrence of metachronous metastases is approximately 25%. Of these, the expected percentage of resected metastases in patients is 10% in the control group and 25% in the intervention group. Given a significance level of 5% and a power of 80%, 115 patients with metastases in both groups are needed. Given an expected recurrence rate of 25%, 460 patients per group are needed.

Because in this study the moment to start the intervention instead of patients will be randomized, we calculated a correction factor C. Assuming a standard deviation in the effect of hospitals on the outcome of 0.1, C has a value of 1.71. (van Houwelingen) Therefore about 800 patients per group are needed.

**7. TREATMENT PERIODS**

*7.1. Control period*:

Outpatient clinic at 3 months after end of treatment (surgery or adjuvant chemotherapy)

Assessment for fitness to undergo metastasectomy[[2]](#footnote-3)

Care as usual according to the Dutch guidelines ([www.oncoline.nl](http://www.oncoline.nl/)) and local amendment. The actual care as usual given may differ per hospital. Herefore, the standard care as usual per hospital will be described.

Filling out the questionnaire “SF-12 “ after 12 months

*7.2. Intervention period*:

Outpatient clinic at 3 months after end of treatment (surgery or adjuvant chemotherapy)

Assessment for fitness to undergo metastasectomy^2^

During year 1

CEA measurement every 8 weeks at local laboratory, report results by letter. The possibilities for blood sampling at a local laboratory are now examinated per hospital. The measurement will take place at the hospital itself, samples will be mailed to the laboratory.

If no special events occur, no additional out patient clinic visits

At year 1 helical CT-abdomen[[3]](#footnote-4) and colonoscopy[[4]](#footnote-5)

Filling out the questionnaire “SF-12 “ after 12 months

Filling out the questionnaire of intensified follow-up at 1 year

One year after surgery outpatient clinic visit

- Results from evaluation (CT) and evaluation long term surgical results

- Update assessment for fitness metastasectomy

During year 2

CEA measurement every 8 weeks at local laboratory, report results by letter

If no special events occur, no additional out patient clinic visits

At year 2 helical CT thorax/abdomen^4^

Two years after surgery outpatient clinic visit

Results from evaluation (CT) and evaluation long term surgical results

Update assessment for fitness metastasectomy

During year 3

CEA measurement every 12 weeks at local laboratory, report results by letter

If no special events occur, no additional out patient clinic visits

At year 3 helical CT thorax/abdomen

Three years after surgery outpatient clinic visit

Results from evaluation (CT) and evaluation long term surgical results

Update assessment for fitness metastasectomy

All patients will be informed about the laboratory results by letter or email. In case of positive results patients will be invited to undergo a CT scan followed by an outpatient clinic visit. All patients will have access to the surgeon’s outpatient clinic in case of questions or clinical complaints.

*7.2.1.Procedure when CEA crosses the threshold value (see flowchart, addendum 2)*

Increase > 20% in 8 weeks > repetition of CEA measurement in 4 weeks, also measuring CRP level.

Increase in second measurement after 4 weeks > 10%, with a lower static threshold value of 2.5 ng/ml > outpatient clinic visit; if explanation of rise is clear from clinical reasons (ie., active infection): treat infection and re-assess CEA value after 4 weeks. If increase:

-> Evaluation by helical CT Thorax/abdomen within two weeks:

-> Outpatient clinic within 7 days after CT scan

At this visit an assessment is made evaluating

- The presence of resectable metastasis in liver or lung

- Physical signs suggesting other localizations of recurrent disease

- Fitness of the patient to undergo treatment of metastasis

When curable metastases are found and the patient can undergo metastasectomy

-> Treatment at own or referral centre according to the current standard

When irresectable metastases are found or the patient is unfit to undergo metastasectomy

-> Referral to medical oncology for palliative chemotherapy according to the current standard;

-> Referral to radiotherapy when palliative radiation is clinically considered

When no liver and lung metastases are found on CT scan

-> CEA measurement as before

-> Next step dependent on outcome, to discuss in interdisciplinary platform

## 8. METHODS

## *8.1. Study parameters/endpoints*

### *8.1.1. Main study parameter/endpoint*

Percentage of patients with curative metastasectomy of liver- and lung metastasis from colorectal carcinoma in the study period: July 2010 – October 2012.

### *8.1.2. Secondary study parameters/endpoints*

Calculation of the optimal threshold values and measurement frequency of CEA by

means of calculating ROC curves based on actually measured CEA values.

Percentage of patients that show metastasis on CT thorax/abdomen when the rise of CEA at the chosen threshold values is above normal, and percentage of patients that do not show metastasis on CT thorax/abdomen despite rise of CEA.

Percentage of patients that show metastasis on regular CT Thorax/abdomen with no rise of CEA above normal (false negative rate of CEA measurement)

Overall survival, disease-free survival and recurrence-free survival

### *8.1.3. Other study parameters*

Co-morbidity influencing CEA: Smoking, benign gastro-intestinal disorders

Incidence of second primary malignancies that cause a rise in CEA

Patient characteristics

Tumour characteristics

Pre-operative CEA and CT thorax/abdomen (cTNM stage)

## *Study procedures*

## Vena puncture bi-monthly: measurement serum CEA and serum ‘in storage’

## Vena punctures are carried out at local laboratories.

## CT scan abdomen/thorax

## At year 1 (abdomen) and 2 (thorax/abdomen)

## If CEA rises as outlined in the flowchart, a CT scan thorax/abdomen will be performed because of the suspicion for local recurrence or metastatic disease. If this scan is made coincidently at or around year 1, 2 or 3, the standard scan will be cancelled.

## *8.3. Withdrawal of individual subjects*

Subjects can leave the study at any time for any reason if they wish to do so without any consequences. The investigator can decide to withdraw a subject from the study for urgent medical reasons.

## *8.4. Replacement of individual subjects after withdrawal*

Not applicable

## *8.5. Follow-up of subjects withdrawn from study*

Patients withdrawn from the study will be treated according to the common clinical practice (that is, regular follow-up) and their characteristics (incidence of metastasis, curative treatment) will be followed. They will be asked for reasons from withdrawal to gain insight in possible undesired effects of the intensified follow-up protocol.

## *8.6 Premature termination of the study*

## Premature termination of the study will be carried out when the follow-up according to the current guideline cannot be guaranteed.

## 9. SAFETY REPORTING

## *9.1. Section 10 WMO event*

In accordance to section 10, subsection 1, of the WMO, the investigator will inform the subjects and the reviewing accredited METC if anything occurs, on the basis of which it appears that the disadvantages of participation may be significantly greater than was foreseen in the research proposal. The study will be suspended pending further review by the accredited METC, except insofar as suspension would jeopardize the subjects’ health. The investigator will take care that all subjects are kept informed.

## *9.2. Adverse and serious adverse events*

Adverse events are defined as any undesirable experience occurring to a subject during a clinical trial. All adverse events reported spontaneously by the subject or observed by the investiga­tor or his staff will be recorded.

A serious adverse event is any untoward medical occurrence or effect that at any dose results in death;

- is life threatening (at the time of the event);
- requires hospitalization or prolongation of existing inpatients’ hospitalization;
- results in persistent or significant disability or incapacity;
- is a congenital anomaly or birth defect;
- is a new event of the trial likely to affect the safety of the subjects, such as an unexpected outcome of an adverse reaction, lack of efficacy of an IMP used for the treatment of a life threatening disease, major safety finding from a newly completed animal study, etc.

All SAEs will be reported to the accredited METC that approved the protocol, according to the requirements of that METC.

Anticipated adverse events

- Haematoma from vena puncture
- Anxiety caused by frequent vena punctures

## *9.3. Follow-up of adverse events*

All adverse events will be followed until they have abated, or until a stable situation has been reached. Depending on the event, follow up may require additional tests or medical procedures as indicated, and/or referral to the general physician or a medical specialist.

## *9.4. Data Safety Monitoring Board (DSMB)*

Not applicable

# 10. STATISTICAL ANALYSIS

In the clinical study, the main analysis will be based on the differences between care as usual (pre-treatment) and care after implementation of the CEA watch (post-treatment) regarding the percentage of patients with resected metastases, using a multilevel model to adjust for the clustering. For the quality of life data the same analysis will be applied. In addition, potential confounders such as age, gender and tumour status will be considered.

In an economic evaluation, costs and outcomes of both follow-up strategies will be assessed and compared. Research question in the economic evaluation: “What is the incremental cost-effectiveness of a computer based (CEA-watch) follow-up protocol, compared to follow-up care as usual?” Results of the cost-effectiveness analysis will display the additional costs per additional patient with curative metastasectomy. In addition, a cost-utility analysis will be conducted, based on the quality adjusted life years (QALYs). Both types of analyses will be conducted in order to be able to estimate the balance between costs and effects on health, survival and quality of life. Costs will be studied from a societal perspective, during a time period maximally 3 years. Since the time horizon of the study exceeds one year, both costs and effects beyond the first year will be discounted, using various discounting rates of 3%, 4% and 5%, and no discounting, to facilitate international comparison of study results.

Bootstrapping will be performed on the cost and effect pairs in order to calculate confidence intervals. Cost effectiveness acceptability curves will be constructed to illustrate the probability that the different follow-up strategies will be cost-effective, given different monetary values per additional unit of health (patient with patient with curative metastasectomy/QALY).

Relevant cost components of both follow-up strategies will be assessed for all included patients. Main cost categories will be ultrasound, CT-scan, colonoscopy, CEA assay, ‘CEA watch’ system, additional diagnostic tests, outpatient visits, hospital admissions and travel expenses.

Costs of productivity losses will not be taken into account, since the target population largely consists of retired people. Unit costs will be valued using Dutch standard prices. In case no standard prices are available, costs will be calculated according to the Dutch guidelines for cost studies (CVZ) . Volumes on cost categories will be registered on a patient level, except for travelling expenses, for which a question will be added to the Quality of Life questionnaire.

# 11. ETHICAL CONSIDERATIONS

## *11.1. Regulation statement*

The study will be conducted according to the principles of the Declaration of Helsinki and in accordance with the Medical Research Involving Human Subjects Act (WMO) and according to the ICH-GCP guidelines

## *11.2. Recruitment and consent*

All patients that underwent curative resection of colorectal carcinoma will be recruited on the first outpatient clinic visit, either by the study coordinator or responsible surgeons. They will be informed about the study and asked permission for participation. They are given 1 month decision time. The patient must give written informed consent before any study related procedures will be initiated. The alternative will be the local follow-up guideline.

## *11.3. Objection by minors or incapacitated subjects*

## Not applicable

## *11.4. Benefits and risks assessment, group relatedness*

## The potential value from this study is an increase in survival due to a more effective follow-up protocol. When this goal is achieved, the participating patients will directly benefit from their participation. When this goal is not achieved, the participation does not substantially harm the participating patients, though some increase in concern about possible recurrence might occur due to the frequent bloodtests.

## *11.5. Compensation for injury*

Since this intensified follow-up scheme does not contain any risks for patients except the small risk for suffering a haematoma after vena puncture, dispensation for Insurance of patients is requested at the Medical-Ethical Committee.

## *11.6. Incentives*

No incentives for participation are received by the patients.

**12.ADMINISTRATIVE ASPECTS AND PUBLICATION**

## *12.1. Handling and storage of data and documents*

All data will be kept in the DSCA database and will include the patients full name and address for study purposes during the clinical trial. Access is granted to participating surgeons, the study coordinator and database technical manager. All additional data will be kept online until the study is ended, after which the data will be stored by the study coordinator. The subjects privacy is protected through secure links and limited access.

The serum samples will be kept in storage for maximum of 10 years.

## *12.2. Amendments*

Amendments are changes made to the research after a favorable opinion by the accredited METC has been given. All amendments will be notified to the METC that gave a favorable opinion.

## *12.3. Annual progress report*

The investigator will submit a summary of the progress of the trial to the accredited METC once a year. Information will be provided on the date of inclusion of the first subject, numbers of subjects included and numbers of subjects that have completed the trial, serious adverse events/ serious adverse reactions, other problems, and amendments.

## *12.4. End of study report*

The investigator will notify the accredited METC [and the competent authority] of the end of the study within a period of 8 weeks. The end of the study is defined as the last patient’s last visit. In case the study is ended prematurely, the investigator will notify the accredited METC [and the competent authority within 15 days], including the reasons for the premature termination. Within one year after the end of the study, the investigator will submit a final study report with the results of the study, including any publications/abstracts of the study, to the accredited METC [and the Competent Authority].

## *12.5. Public disclosure and publication policy*

Results of the study will be published without restriction; no arrangements are applicable concerning disclosure of research data with the sponsor.

# 13. REFERENCES

Bentrem DJ, DeMatteo RP, Blumgart LH. Surgical therapy for metastatic disease to

the liver. Annu Rev Med 2005; 56: 139-156

Boey J, Cheung HC, Lai CK, Wong J. A prospective evaluation of serum

carcinoembryonic antigen (CEA) levels in the management of colorectal carcinoma.

World J Surg 1984; 8: 279-286

Borie F, Daurès JP, Millat B, Trétarre B. Cost and effectiveness of follow-up examinations

in patients with colorectal cancer resected for cure in a French population-based study. J Gastrointest Surg. 2004 Jul-Aug;8(5):552-8.

Cooper GS, Kou TD, Chak A. Receipt of previous diagnoses and endoscopy and outcome

from esophageal adenocarcinoma: a population-based study with temporal trends. Am J Gastroenterol. 2009 Jun;104(6):1356-62.

Gold P, Freedman SO. Specific carcinoembryonic antigens of the human digestive system.

J Exp Med. 1965 Sep 1;122(3):467-81.

Goldberg RM, TR Fleming, CM Tangen e.a. Surgery for recurrent colon cancer:

strategies for identifying resectable recurrence and success rates after resection.

Ann Intern Med 1998; 129: 27-35

Graham RA, Wang S, Catalano PJ, Haller DG. Postsurgical surveillance of colon

cancer: preliminary cost analysis of physician examination, carcinoembryonic antigen

testing, chest X-ray and colonoscopy. Ann Surg 1998; 228: 59-63

Grossmann EM (b), FE Johnson, KS Virgo, WE Longo, R Fossati. Follow-up of

colorectal cancer patients after resection with curative intent – the GILDA trial. Surg

Oncol 2004: 13; 119-124

Grossmann I (a), G.H. de Bock, C.J.H. van de Velde, J. Kievit, T. Wiggers. Results of a

national survey among Dutch surgeons treating patients with colorectal carcinoma.

Current opinion about follow-up. Colorectal Disease 2007; 9: 787-792

Grossmann I (c), G.H. de Bock, W.M. Meershoek-Klein Kranenbarg, C.J.H. van de

Velde, T. Wiggers. CEA measurement during follow-up for colorectal carcinoma is

useful even if normal levels exist before curative surgery. Eur J Surg Oncol 2007: 33;

183-187

Houwelingen JC van. Randomisatie op het niveau van behandelaars. Ned Tijdschr

Geneeskd 1998; 142: 1662-5

Kobayashi H. Characteristics of recurrence and surveillance tools after curative

resection for colorectal cancer: a multicentre study. Surgery 2007: 141: 67-75

Minton JP, hoehn JL, Gerber DM, Horsley JS, Connoly DP, salwan F, Fletcher WS,

Cruz AB, Gatchell FG, Oviedo M, Meyer KK, Leffal LD, Berk RS, Stewart PA, Kurucz

SE. Results of a 400-patient carcinoembryonic antigen second-look colorectal cancer

study. Cancer 1985; 55: 1284-1290

Pfannschmidt J, H Dienemann, H Hoffmann. Surgical resection of pulmonary

metastases from colorectal cancer: a systematic review of published series. Ann

Thorac Surg 2007; 84: 324-338

Spratlin JL, D Hui, J Hanson, C Butts HJ Au. Community compliance with

carcinoembryonic antigen: follow-up of patients with colorectal cancer. Clin

Colorectal Cancer 2008; 7: 118-125

Staab HJ, Anderer FA, Stumpf E, Hornung A, Fischer R, Kieniger G. Eighty-four

potential second-look operations based on sequential carcinoembryonic antigen

determinations and clinical investigations in aptients with recurrent gastro-intestinal

cancer. Am J Surg 1985; 149: 198-204

Staab HJ, Anderer FA. Circulating carcinoembryonic antigen (CEA), a growth

parameter in malignant disease. Canc Detect Prev 1983; 6: 33-39

Staab HJ, Anderer FA, Hornung A, Stumpf E, Fischer R. Doubling time of circulating

CEA and its relation to survival of patients with recurrent colorectal cancer. Brit J

Cancer 1982: 46: 773-781

Staab HJ, Anderer FA, Stumpf E, Fischer R. Slope analysis of the post-operative

CEA time course and it’s possible application as an aid in diagnosis of disease

progression in gastro-intestinal cancer. Am J Surg 1978; 136: 322-327

Steele G, Ellenberg S, Ramming K, O’Connell M, Moertel C, Lessner H, Bruckner H,

Horton J, Schein P, Zamcheck N, Novak J, Holyoke ED. CEA monitoring among

patients in multi-institutional adjuvant G.I. therapy protocols. Ann Surg 1982: 196 (2): 162-169

Sugarbaker PH, Gianola FJ, Dwyer A, et al. A simplified plan for follow-up of patients

with colon and rectal cancer supported by prospective studies of laboratory and radiological test results. Surgery 1987;102:79–87.

National working group of gastro-intstinal tumours. Dutch National Guidelines on

colon cancer (version 2.0) and rectal cancer (version 2.1). http://www.oncoline.nl/

National Cancer Institute.

- http://www.cancer.gov/cancertopics/pdq/treatment/colon/HealthProfessional/page2

- <http://www.cancer.gov/cancertopics/pdq/treatment/rectal/HealthProfessional/page2>

- http://CEAwatch.umcg.nl

1. Curative treatment is based on pre-operative evaluation (imaging of chest and abdomen) and radical treatment as confirmed by operation and pathology report. Adjuvant chemotherapy is given according to the current standard. [↑](#footnote-ref-2)
2. Only when the patient is fit enough for metastasectomy, follow-up is started. This is assessed at the first outpatient visit and if necessary delayed at the surgeon’s judgment. [↑](#footnote-ref-3)
3. When a patient underwent a CT thorax/abdomen due to a rise in CEA during follow-up, the study CT at year 1 and 2 will not be made. [↑](#footnote-ref-4)
4. When a full colonoscopy has been performed pre-operatively, the colonoscopy at year 1 is not indicated. [↑](#footnote-ref-5)
